# Screening and identifying natural products with SARS-CoV-2 infection inhibitory activity from medicinal fungi

**DOI:** 10.1016/j.bsheal.2023.12.006

**Published:** 2023-12-31

**Authors:** Shuang Zhao, Amelia Siqi Huang, Weibo Zhang, Lili Ren, Hexiang Wang, Jianbin Wang, Xinyang Shao, Guanbo Wang

**Affiliations:** a Institute of Agri-Food Processing and Nutrition, Institute of Plant Protection, Beijing Academy of Agriculture and Forestry Sciences, Beijing 100097, China; b Beijing Key Laboratory of Fruits and Vegetable Storage and Processing, Key Laboratory of Vegetable Postharvest Processing, Ministry of Agriculture, Beijing 100097, China; c Dalton Academy, The Affiliated High School of Peking University, Beijing 100190, China; d NHC Key Laboratory of Systems Biology of Pathogens and Christophe Mérieux Laboratory, National Institute of Pathogen Biology, Beijing 100730, China; e Key Laboratory of Respiratory Disease Pathogenomics, Chinese Academy of Medical Sciences and Peking Union Medical College, Beijing 100730, China; f College of Biological Sciences, China Agricultural University, Beijing 100193, China; g School of Life Sciences, Tsinghua University, Beijing 100084, China; h Changping Laboratory, Beijing 102206, China; i Institute for Cell Analysis, Shenzhen Bay Laboratory, Shenzhen 518132, China

**Keywords:** Medicinal fungi, Natural products, Antiviral activity, COVID-19, Polysaccharides

## Abstract

The coronavirus disease of 2019 (COVID-19), a global pandemic caused by the severe acute respiratory syndrome coronavirus 2 (SARS-CoV-2), can result in severe health complications. In addition to physical preventative measures, pharmaceutical intervention is also crucial. Numerous natural products from medicinal fungi have shown promise as potential antiviral drugs and may serve as a source of effective components with antiviral activity against SARS-CoV-2 and other coronaviruses. In this study, we developed a workflow that integrates viral infection inhibition assays at both cellular and molecular levels, as well as molecular separation and characterization, to screen and identify natural products with antiviral activity. Using this workflow, we screened 167 extracts extracted from 36 medicinal fungi using optimized extraction methods. We assessed the antiviral effects of these extracts by measuring their ability to inhibit SARS-CoV-2 infection and receptor binding domain - human angiotensin-converting enzyme 2 (RBD-hACE2) binding *in vitro*. Following charge- and size-based characterization of the active compounds through filtration and chromatographic fractionation, mass spectrometry characterization of the fractionated compounds revealed that the active components are polysaccharides and determined their monosaccharide residue composition. Our findings provide new insights into the antiviral potential of natural products and their screening strategies and may contribute to the development of effective antiviral therapeutics against COVID-19 and other diseases.

## Highlights

• Scientific questions: Despite the rapid development of vaccines and therapeutics, the quest for efficacious and safe anti-coronavirus disease 2019 (COVID-19) agents, especially those derived from natural sources, remains a pressing concern. The criticality of a rational screening design that bridges functional tests with *in vivo* experiments, coupled with reliable structural characterization, cannot be overstated in the exploration of the antiviral effects of natural products.
• Evidence before this study: Previous research has underscored the antiviral and anti-inflammatory properties of medicinal fungi and their derivatives against a variety of respiratory viruses. The successful drug development based on marine fungi illuminates the potential of terrestrial fungi in this domain. Their wide-ranging antiviral activity, immunomodulatory effects, and diverse chemical constituents position them as promising candidates for further exploration.
• New findings: We developed a workflow to screen and identify natural products with anti-COVID activity at both cellular and molecular levels. We screened 167 extracts derived from 36 medicinal fungi using optimized extraction methods. Our findings indicate that polysaccharides from certain fungi exhibit potent inhibitory effects on the severe acute respiratory syndrome coronavirus 2 (SARS-CoV-2) infection and the receptor binding domain - human angiotensin-converting enzyme 2 (RBD-hACE2) binding.
• Significance of the study: This study sheds new light on the antiviral potential of natural products and their screening strategies. It holds promise for contributing to the development of effective antiviral therapeutics against COVID-19 and potentially other diseases.

## Graphical abstract

**Figure:**
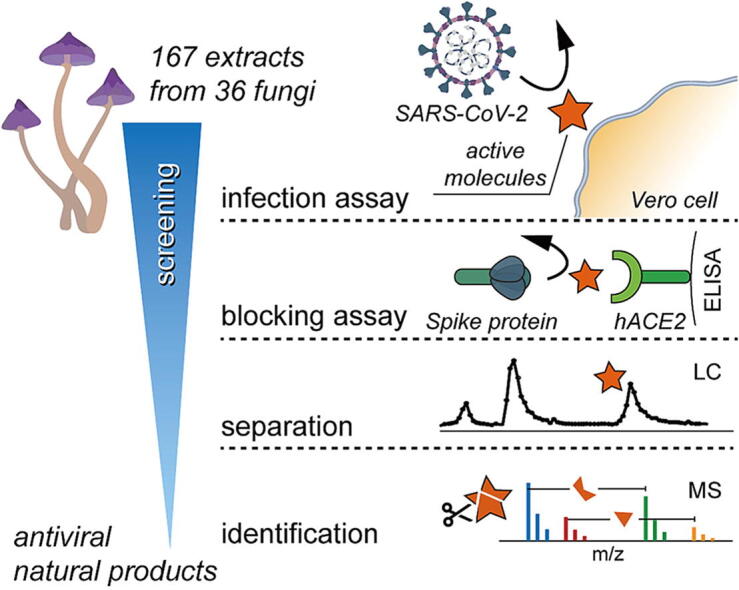
Employing a comprehensive workflow that incorporates both cellular and molecular viral infection inhibition assays, we screened 167 extracts derived from medicinal fungi for their potential anti-COVID-19 (coronavirus disease 2019) activity. The active compounds, identified as polysaccharides, were characterized using an array of molecular separation techniques and mass spectrometry

## 1 Introduction

The coronavirus disease 2019 (COVID-19) pandemic, caused by the severe acute respiratory syndrome coronavirus 2 (SARS-CoV-2), has posed a serious threat to global public health and socio-economic stability [1]. Despite the rapid development of vaccines and therapeutics, there remains an urgent need for effective and safe anti-COVID-19 agents, particularly from natural sources. Natural products (NPs) have long been a rich source of bioactive compounds for drug discovery, particularly for infectious diseases [2], [3], [4], [5], [6], [7]. NPs can be derived from a variety of sources, including plants [1], animals [5], marine life [8], and fungi [7]. Several studies have demonstrated the antiviral and anti-inflammatory effects of medicinal fungi and their derivatives against various respiratory viruses, including influenza virus [9], [10], respiratory syncytial virus [11], [12], and severe acute respiratory syndrome coronavirus 1 (SARS-CoV-1) [13]. Promising natural products in COVID-19 therapy include but are not limited to cordycepin [14], Lichen Database [5], and BIOFAQUIM [5].

Medicinal fungi have garnered considerable interest due to their diverse chemical constituents and pharmacological activities [6]. While some research has focused on fungi, the majority of these work have centered on marine fungi, which comprise more than 10,000 species [15]. Marine fungi are capable of producing bioactive metabolites *in vivo* or *in vitro* activity against multiple infections [8], representing a new avenue for extracting natural product components from terrestrial fungi. The successful development of drugs based on marine fungi also highlights the potential for drug development from terrestrial fungi. Fungal natural products hold significant potential for the development of anti-COVID-19 drugs. Their broad-spectrum antiviral activity, immunomodulatory effects, and diverse chemical structures make them valuable candidates for further investigation.

A rational design of the screening process is essential for identifying promising drug candidates from fungi. The major screening processes include virtual screening [14], [16], [17], *in vitro* screening at the protein–protein interaction level [18], [19], and cellular functional screening [20]. Virtual screening is a widely used method by drug developers due to its time and cost-saving benefits. The development of computer-aided drug design (CADD) [16], [21], [22] has greatly facilitated structure-based virtual screening (SBVS) and advanced the virtual screening strategy to a new level. Screening at the protein–ligand interaction level is one of the most commonly used *in vitro* methods [13], allowing for the assessment of candidate compounds in real experiments and evaluating their blocking effectiveness using actual proteins rather than virtual computation. For COVID-19 therapeutics, two proteins that played an essential role in the virus’s cell-entry process are the human angiotensin-converting enzyme 2 (hACE2) and the receptor binding domain (RBD) of the spike protein of SARS-CoV-2 [23], [24]. The interaction between these two proteins is key to developing effective drug candidates [25]. Functional screening at the cellular level focuses on cells rather than proteins. This method monitors cellular behaviors and virus reproduction in infected cells treated with candidate compounds in culture medium [13], [15], [26]. Although these approaches offer potential screening criteria, the antiviral effects of fungal natural products are not well explored, as many studies have not connected the functional tests with *in vivo* experiments. Thus, it is crucial to integrate and coordinate these strategies to achieve conclusive results.

In this study, we aimed to investigate the potential of medicinal fungal extracts as inhibitors of SARS-CoV-2 infection. We optimized and assessed the extraction methods of 36 medicinal fungi, producing 167 extracts. These extracts were then tested for their inhibitory effects using various assays, including viral infection of Vero cells, protein binding inhibition, active molecule fractionation, and toxicity evaluation. Our results confirmed that the aqueous extract of *Inonotus obliquus* (*I. obliquus*) and *Pholiota adiposa* (*P. adiposa*) could prevent viral infection by blocking the binding between the viral Spike protein RBD and hACE2 proteins. The extracts showed promising safety profiles, with no observable acute toxicity in mice. Fractionation studies of *l. obliquus* through centrifugal filtration and chromatographic elution indicated that the active components were likely to be polysaccharides, a finding that was verified by mass spectrometry (MS) results. These findings offer new insights into the antiviral potential of natural products from medicinal fungi.

## 2 Materials and methods

### 2.1 Materials and reagents

*I. obliquus* was purchased from Jilin Xiaobei Biotechnology Co. *Trametes robiniophila* (*T. robiniophila*, also known as Huaier), *P. adiposa* and *Stropharia rugosoannulata* (*S. rugosoannulata*) were obtained from the Institute of Agri-Food Processing and Nutrition, Beijing Academy of Agriculture and Forestry Sciences. *Phellinus igniarius* (*P. igniarius*), cultured under various conditions (such as bag or log culture), was kindly gifted by Prof. Yajie Zou from the Institute of Agricultural Resources and Agricultural Regional Planning at the Chinese Academy of Agricultural Sciences. *Agaricus bisporus* (*A. bisporus*), *Hypsizygus marmoreus* (*H. marmoreus*), *Agrocybe cylindracea* (*A. cylindracea*), *Panus gigianteus* (*P. gigianteus*), *Lentinus edodes* (*L. edodes*) and *Ganoderma Lucidum* (*G. Lucidum*) were purchased from a local market in Beijing. *Dictyophora indusiata* (*D. indusiata*), *Ramaria botrytoides* (*R. botrytoides*), *Tricholoma matsutake* (*T. matsutake*), *Tuber melanosporum* (*T. melanosporum*), and *Cordyceps sinensis* (*C. sinensis*) and other fungi listed in Supplementary Table S1 were purchased from a local market in Yunnan. Resorcinol was purchased from Solabo (Beijing), and DE-52 from Coolab (Beijing). An anti-SARS-CoV-2 neutralizing antibody kit based on enzyme-linked immunosorbent assay (ELISA), EKnCov001 from Frdbio (Wuhan, China), was employed to assess the inhibition effectiveness of each sample. Trypsin (Cat. # 25200072) was purchased from Thermo Fisher. Other common reagents were obtained from China Pharma or Beijing Chemical Industry Co.

### 2.2 Extraction

Crude extracts were obtained from dried fungi through extraction. Using *I. obliquus* as an example, the fruiting bodies of the fungi were powdered using a small food processor at room temperature. The powder was then added to deionized water or other specified solvents in a mass ratio of 1:40 and subjected to a water bath at 50 °C for 4 h. The mixture was centrifuged at 8,000 rpm (5430R, Eppendorf, Germany) for 30 min, and the supernatant was filtered through a 0.22 µm filter. The filtered solution was placed in a new 50 mL centrifuge tube with the cap open in an oven at 50 °C overnight to evaporate most of the solvent. The remaining solution was transferred to microcentrifuge tubes and dried in a centrifugal evaporator (Concentrator Plus, Eppendorf) under vacuum at 45 °C for 24 h to obtain crude extract. A similar protocol was followed by other fungi.

### 2.3 Cell culture

Vero cells (ATCC) were cultured in high-glucose Dulbecco's modified eagle medium (DMEM) (C11995500BT, Gibco, USA) supplemented with 10% fetal bovine serum (35-081-CV, Corning, USA) at 37 °C with 5% CO_2_ in an incubator. The day before the experiment, Vero cells were seeded at a density of 10,000 cells per well in a 96-well plate.

### 2.4 SARS-CoV-2 infection

The SARS-CoV-2 coronavirus was isolated from clinical samples in Vero cells in a BSL-3 laboratory. For the half maximal inhibitory concentration (IC50) test, a 96-well deep-well plate was used to dilute the drugs up to a 1:1,000 ratio, with 3-fold steps. Remdesivir (initial concentration of 10 μmol/L) and dimethyl sulphoxide (DMSO) were used as positive and negative controls, respectively. After washing Vero cells with a blank DMEM, 100 µL of the sample solution was added to each well and incubated at 37 °C for 1 h. Then, SARS-CoV-2 virus (multiplicity of infection (MOI) = 0.1) was added and incubated at 37 °C for another 1 h. After incubation, the cells were washed with 200 µL of blank DMEM, and then 100 µL of the sample solution was added and continued to incubate at 37 °C for another 23 h. Three wells were used for each concentration. After incubation, 80 µL of supernatant was taken and mixed with 300 µL of Trizol LS (Direct-zol RNA MiniPrep kit, R2052, Zymo Research) for nucleic acid extraction following the manufacturer’s instructions. We used quantitative reverse transcription polymerase chain reaction (PCR) to detect the copy number of the *N* gene of the SARS-CoV-2 virus [27].

### 2.5 ELISA assay

The assay was performed following the manufacturer’s protocol. The microtiter plate was covalently coated with RBD of SARS-CoV-2 Spike protein. Samples were added to the plate wells along with biotinated hACE2 (Bio-hACE2), which was then bound to streptavidin-conjugated horseradish peroxidase (HRP). A colorimetric ELISA was carried out by detecting the enzymatic conversion of 3,3',5,5'-tetramethylbenzidine (TMB). Briefly, the process could be divided into six sections. First, the sample solution was diluted with universal dilution buffer at a volume ratio of 1:9, and the positive and negative controls were diluted with universal dilution buffer at a volume ratio of 1:99. All diluted solutions were added to the plate wells, 50 µL each. Second, 50 µL Bio-ACE2 solution was added to each well and mixed gently with a shaker (MS3, IKA, Germany). The wells were sealed with a plate laminate sealer and incubated at 37 °C for 60 min. Third, the sealer was removed and the wells were washed three times with 350 µL of washing buffer. After washing, the wells were patted dry on absorbent paper. Fourth, 100 µL of HRP-Streptavidin solution was immediately added to each well and mixed gently with a shaker. The wells were sealed and incubated at 37 °C for 60 min. Fifth, the sealer was removed and the wells were washed three times with 350 µL of washing solution. After washing, the wells were patted dry on absorbent paper. Finally, 100 µL of TMB substrate solution was immediately added to each well, causing the solution to turn blue. After waiting for 10–15 min at room temperature, 50 µL of substrate reaction stop solution was added to each well once the color had stabilized. We then used a plate reader (Spark 20 M, Tecan, Switzerland) to measure the absorbance of each well at a wavelength of 450 nm, with 630 nm as a reference.

Inhibition efficiency was calculated from either viral infection experiments or by the ELISA tests at the molecular level. Through cell infection experiments, the infection efficiency was defined as viral load and calculated as

(1) $$1-\frac{\overset{¯}{V_{\mathrm{Exp}}}}{\overset{¯}{V_{C}}})\times 100\%$$

where *V*_*Exp*_ represents viral load, as reflected by the amount of viral genome RNA measured by quantitative reverse transcription PCR experiments; *V*_*C*_ represents viral load in the control group that uses DMSO instead of extracts. Through ELISA tests, inhibition capability was assessed by competitive inhibition of SARS-CoV-2 Spike protein RBD and ACE2 interaction. Inhibition efficiency was calculated as

(2) $$\frac{{\mathrm{OD}}_{\mathrm{NC}}-{\mathrm{OD}}_{S}}{{\mathrm{OD}}_{\mathrm{NC}}}\times 100\%$$

where optical density (*OD*) represents the optical density (absorbance) measured at 450 nm, *NC* represents negative control, and *S* represents sample.

### 2.6 Heat treatment

The extracts of *I. obliquus and P. igniarius* at a concentration of 1 mg/mL, and *T. robiniophila* at a concentration of 250 µg/mL, were subjected to boiling, which resulted in denaturation of the protein components within the extracts. Following the heat treatment, the extracts were allowed to cool and subsequently collected for further analysis. The inhibition efficiency of these extracts before and after boiling was evaluated using the method described in section 2.5 for comparison.

### 2.7 Acute toxicity test

Animal experiments in this study were conducted in compliance with approved protocols and guidelines from the Institutional Animal Care and Use Committee of Beijing Viewsolid Biotechnology Co. LTD (license number: VS2126A00150). The toxicity of fungi extracts was assessed in non-infected male C57 mice, aged eight weeks. A total of 40 mice were randomly assigned into four groups: three test groups and one control group. Each group was further divided into low-dose and high-dose subgroups, with each subgroup consisting of five mice. On Day 1, all mice were administered 0.5 mL of either fungi extract (at a concentration of 25 mg/mL for the text groups) or saline (for the control group) via intragastric administration once (for the low-dose subgroups) or three times (for the high-dose subgroups). Over the course of 15 days, the mice were continuously monitored for changes in viability, behavior, diet, and excretion, and their body weights were measured on Day 1, Day 7, and Day 11.

### 2.8 Liquid chromatography (LC)

A DE-52 anion exchange column (1 cm × 30 cm) was packed and equilibrated with deionized water. The crude extract was fully dissolved in deionized water, and the supernatant was collected after centrifugation at 8,000 rpm for 30 min for loading onto the column. Sequential elution was performed using 0 mmol/L, 500 mmol/L, and 2 mol/L NaCl solutions. Fractions containing active compounds were further purified by size exclusion chromatography (SEC) using a Superdex 200 column (GE Healthcare) on an ÄKTA pure system (GE Healthcare). The injection volume was 200 µL and the mobile phase was ultrapure water at a flow rate of 0.8 mL/min. The eluate was collected, and the polysaccharide content in each tube of the eluate was determined by the phenol–sulfuric acid method.

### 2.9 Mass spectrometry

Polysaccharide analytes were hydrolyzed in 150 mmol/L HCl and incubated at 60 °C for 2 h. The reaction was then neutralized and terminated with a 150 mmol/L NaOH solution. The hydrolyzed samples were analyzed by matrix-assisted laser desorption ionization–time of flight (MALDI-TOF) MS using a RapifleX MALDI-TOF Tissuetyper (Bruker), with sDHB as the matrix.

### 2.10 Data analysis

Data analysis was conducted using Prism (GraphPad) and Flex Analysis (Bruker Daltonics). Multiple t-tests were performed using the two-stage step-up method, also known as the Benjamini & Hochberg method [28]. The resulting *P*-values were reported by the style guidelines of the *New England Journal of Medicine* (https://www.nejm.org/author-center/new-manuscripts).

## 3 Results

### 3.1 Screening and evaluation of the antiviral activity of medicinal fungal extracts

**Fig. 1:**
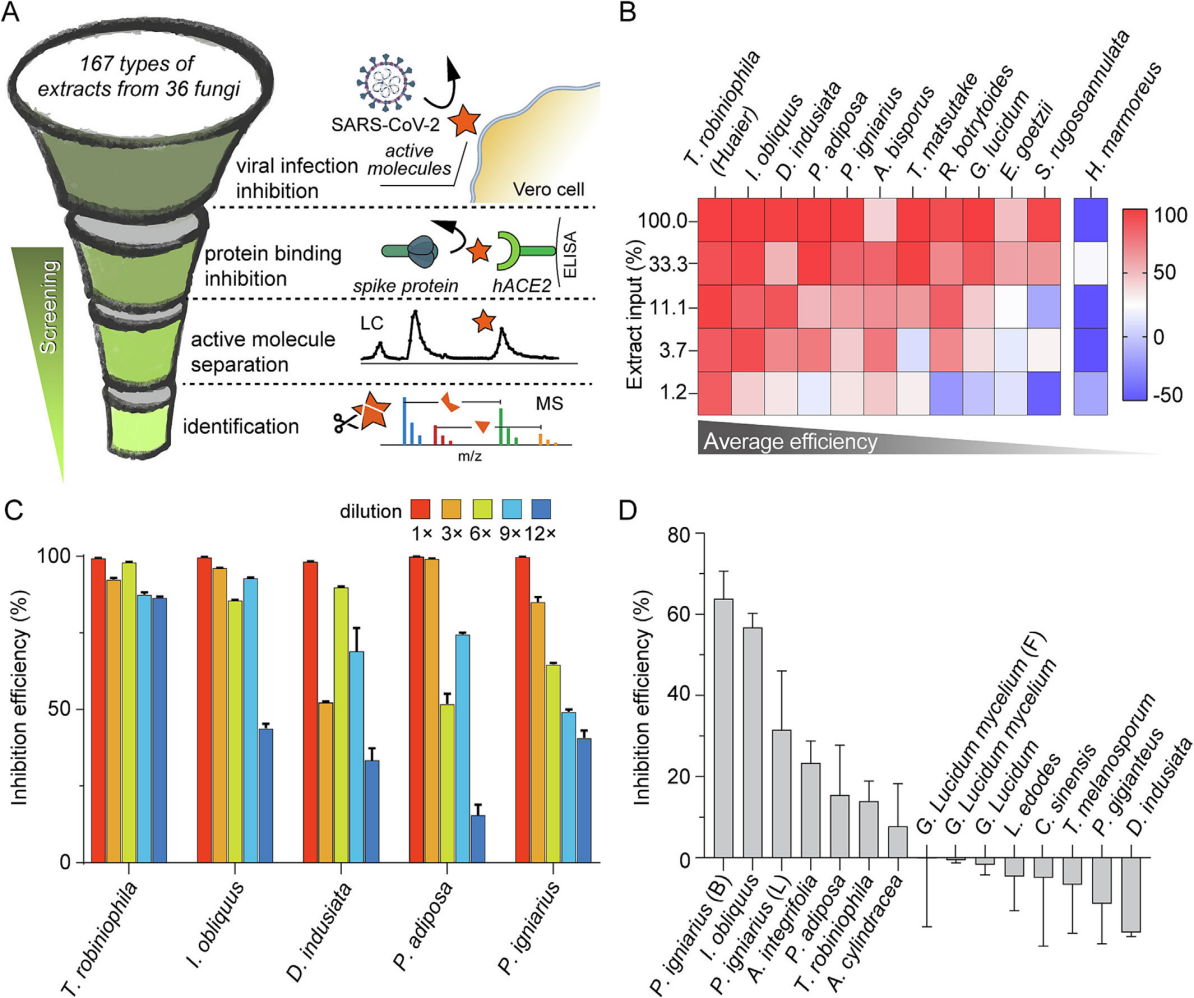
Evaluation of the antiviral activity of medicinal fungal extracts at the cellular and molecular levels. A) Schematic representation of the workflow employed for activity screening, and separation and identification of antiviral compounds derived from medicinal fungi. B) Comprehensive analysis of the antiviral activity exhibited by different fungal extracts against SARS-CoV-2. The viral RNA levels post-cell infection under different extract input quantities were measured to assess the antiviral activity. The inhibition data of *H. marmoreus* extract, which was among the extracts exhibiting no discernable antiviral activity, were incorporated as the reference of negative control. (Initial input quantities: *T. robiniophila* 25 µg for 100% and others 100 µg for 100%). C) Evaluation of the antiviral activity of selected extracts at varying dilutions. (Initial concentrations: *T. robiniophila* 250 µ*g*/mL and others 1 mg/mL). D) ELISA results illustrating the binding inhibition capabilities of the screened extracts. The error bars represent the standard deviation of 3 measurements. Abbreviations: SARS-CoV-2, severe acute respiratory syndrome coronavirus 2; MS, mass spectrometry; hACE2, human angiotensin-converting enzyme 2; ELISA, enzyme-linked immunosorbent assay; *T. robiniophila, Trametes robiniophila; I. obliquus, Inonotus obliquus; P. adiposa, Pholiota adiposa; P. igniarius, Phellinus igniarius; P. gigianteus, Panus gigianteus; H. marmoreus, Hypsizygus marmoreus; A. cylindracea, Agrocybe cylindracea; L. edodes, Lentinus edodes; G. Lucidum, Ganoderma Lucidum; D. indusiata, Dictyophora indusiata; T. melanosporum, Tuber melanosporum; C. sinensis, Cordyceps sinensis*.

We scrutinized 167 extracts from 36 medicinal fungi (Supplementary Table S1) for their potential antiviral activity against SARS-CoV-2, following the workflow outlined in Fig. 1A. The inhibitory performance of these extracts was evaluated using cell infection assays and viral RNA quantification. Vero E6 cells were utilized as the host cells for SARS-CoV-2 infection, and the antiviral efficiency was inferred from the viral RNA levels post-infection. This comprehensive evaluation led to the identification of 11 extracts with notable inhibitory effects on viral infection (Fig. 1B). Despite the detected dependency of inhibition efficiencies on input quantities, certain fungal extracts, namely those from *T. robiniophila*, *I. obliquus*, *D. indusiata*, *P. adiposa,* and *P. igniarius*, exhibited high inhibition efficiencies across a broad range of input quantities. As revealed in the sequential dilution of these promising extracts, the efficiency decreases patterns varied across different extracts due to their distinct components (Fig. 1C).

We further validated the antiviral activity of the candidate extracts through an assay that inhibited the binding of the viral RBD to the hACE2 receptor. We performed ELISA assays to measure the binding inhibition of the previously screened extracts and included additional extracts in the ELISA study to provide more controls for comparison and potentially generate insights into other inhibition mechanisms. As shown in Fig. 1D, the extract of *P. igniarius* cultivated on bags of sawdust (termed as *P. igniarius* (B) in this work), *I. obliquus* and *P. igniarius* cultivated on logs (*P. igniarius* (L)) demonstrated superior performance. The extracts of *P. adiposa* and *A. cylindracea* also exhibited significant inhibition efficiency. The extract of *I. obliquus* and *P. igniarius* showed strong inhibition of interactions between hACE2 and RBD, indicating that blocking this interaction is a key mechanism of their antiviral activity. *P. igniarius* (B), which was cultivated on bags of culture medium based on mulberry sawdust, exhibited higher inhibition efficiency than *P. igniarius* (L), which was cultivated on logs of oak. *D. indusiata*, a candidate that exhibited high inhibition efficiency based on cellular screening, shows the lowest inhibition efficiency in the ELISA assay. This suggests that its inhibition mechanism is unrelated to blocking the RBD-hACE2 interaction. *T. robiniophila*, which has already been the subject of several studies on its inhibitory activity against COVID-19 (Gov. Identifier: NCT04291053), did not exhibit higher inhibitory capabilities than *I. obliquus* or *P. igniarius* for protein binding inhibition (Fig. 1D). These results suggest that *I. obliquus* and *P. adiposa* may be promising choices for extracting molecules with inhibitory activity against SARS-CoV-2 infection.

### 3.2 Characterization of physicochemical properties of the extracts

**Fig. 2:**
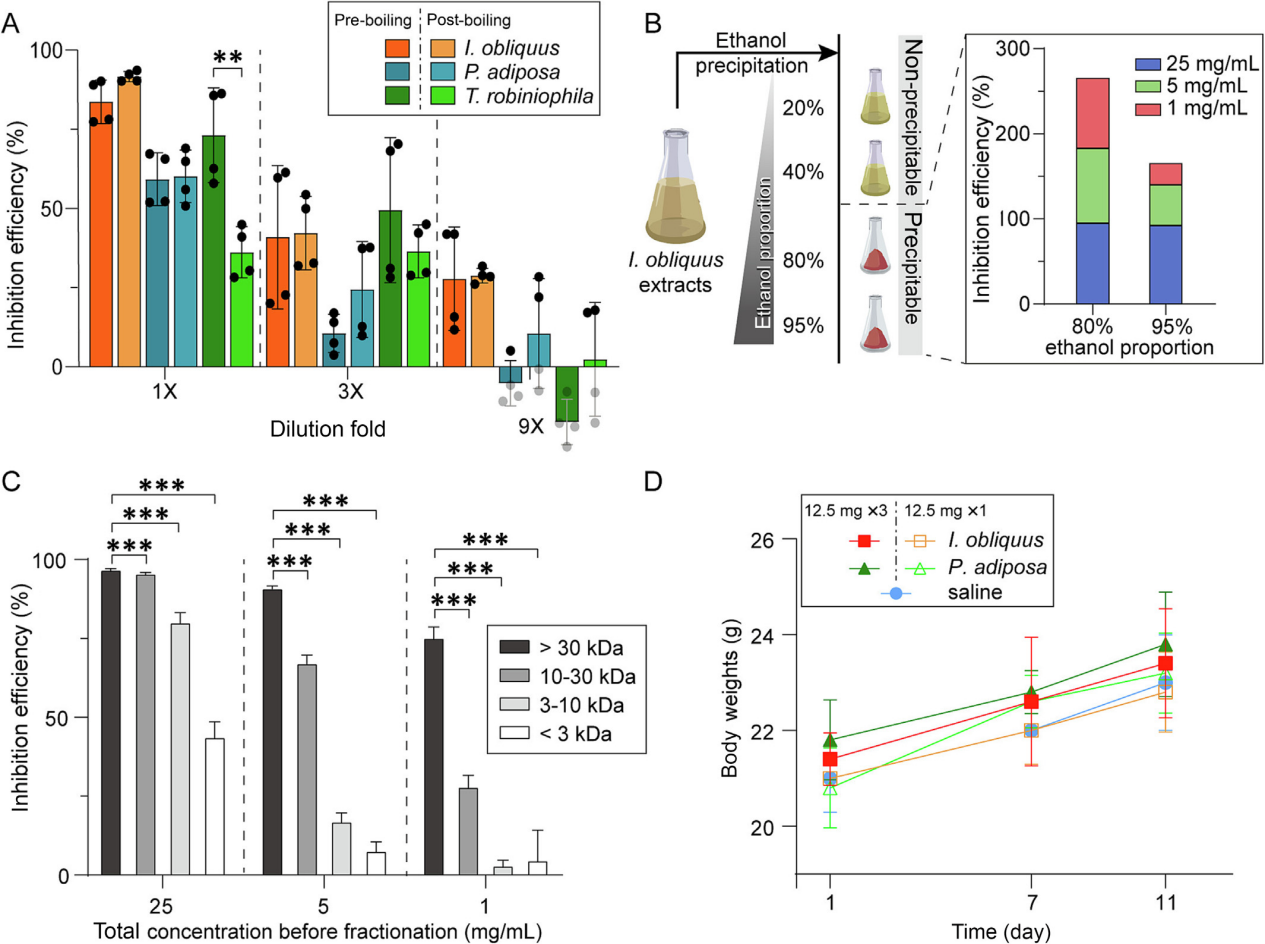
Analysis of physicochemical properties and toxicity of fungi extracts exhibiting potent antiviral activities. A) Comparative analysis of the inhibition efficiency of extracts subjected to heat treatment, conducted to assess the thermostability of antiviral molecules. B) Schematic representation of the ethanol precipitation steps and the antiviral activity of the resulting fractions under ethanol concentrations of 80% and 95%. The fractions were redissolved in water for subsequent activity evaluation. C) Evaluation of the inhibition efficiency of extract fractions from *I. obliquus*, segregated based on varying molecular weight ranges. The error bars represent the standard deviation of 3 measurements. Statistical significance is denoted as follows: * *P* < 0.05, ** *P* < 0.01, *** *P* < 0.001. D) Changes in mouse body weight during an acute toxicity test with different extracts. The mice received 0.5 mL of extracts by oral gavage at a concentration of 25 mg/mL on Day 1. Abbreviations: *I. obliquus, Inonotus obliquus; P. adiposa, Pholiota adiposa; T. robiniophila, Trametes robiniophila*.

We evaluated the thermostability of active molecules from *I. obliquus*, *P. igniarius*, and *T. robiniophila* by subjecting the extracts to boiling (Fig. 2A). The extracts showed distinct stability patterns after boiling. The active components in *I. obliquus* demonstrated high thermostability, with boiling having no discernible impact on the inhibition efficiency. In contrast, for *P. igniarius*, exposure to higher temperatures enhanced the antiviral effectiveness of its extract. However, the activity of the *T. robiniophila* extract was found to be optimal at lower temperatures. These differences in temperature sensitivity suggest that the active components in these fungi may belong to different molecular classes. Notable, the *T. robiniophila* extract lost its activity post-boiling, while the extracts of *I. obliquus* and *P. adiposa* retained their activity. The extracts exhibited comparable performance across different concentrations, suggesting that these natural extracts may contain distinct active components.

We further performed ethanol precipitation to investigate the primary component of the water extract derived from *I. obliquus*. The extract was procured using warm water and subsequently subjected to serial precipitation with escalating concentrations of ethanol (Fig. 2B). No precipitates were formed ethanol concentrations at 20% and 40%. However, higher concentrations of ethanol (80% and 95%) resulted in the formation of precipitates that could be redissolved in water. We then evaluated the inhibition efficiency of these precipitates using an ELISA assay, as previously described. As shown in Fig. 2B, the precipitates obtained from 80% ethanol demonstrated a higher inhibitory efficiency compared to those from 95% ethanol. Notably, even upon dilution, the inhibitory efficiency of the water solution of the precipitates obtained from 80% ethanol remained high.

We fractionated the *I. obliquus* extracts using a centrifugal molecular weight filter and obtained different fractions with varying molecular weight ranges. The inhibitory efficiency of these fractions was evaluated, revealing that molecules exceeding 30 kDa exhibited the highest inhibition activity. Interestingly, the efficiency was observed to decrease as the molecular weight diminished (Fig. 2C). We also performed an acute toxicity test on mice, during which their health conditions, including viability, body weight, behavior, diet, and excretion, were closely monitored. No fatalities were recorded during the test, and the body weights of the mice gradually increased without any significant difference among all experimental groups, including the negative control (Fig. 2D). These results underscore the excellent biosafety of the extracts.

### 3.3 Separation and identification of antiviral polysaccharides from *I. obliquus*

**Fig. 3:**
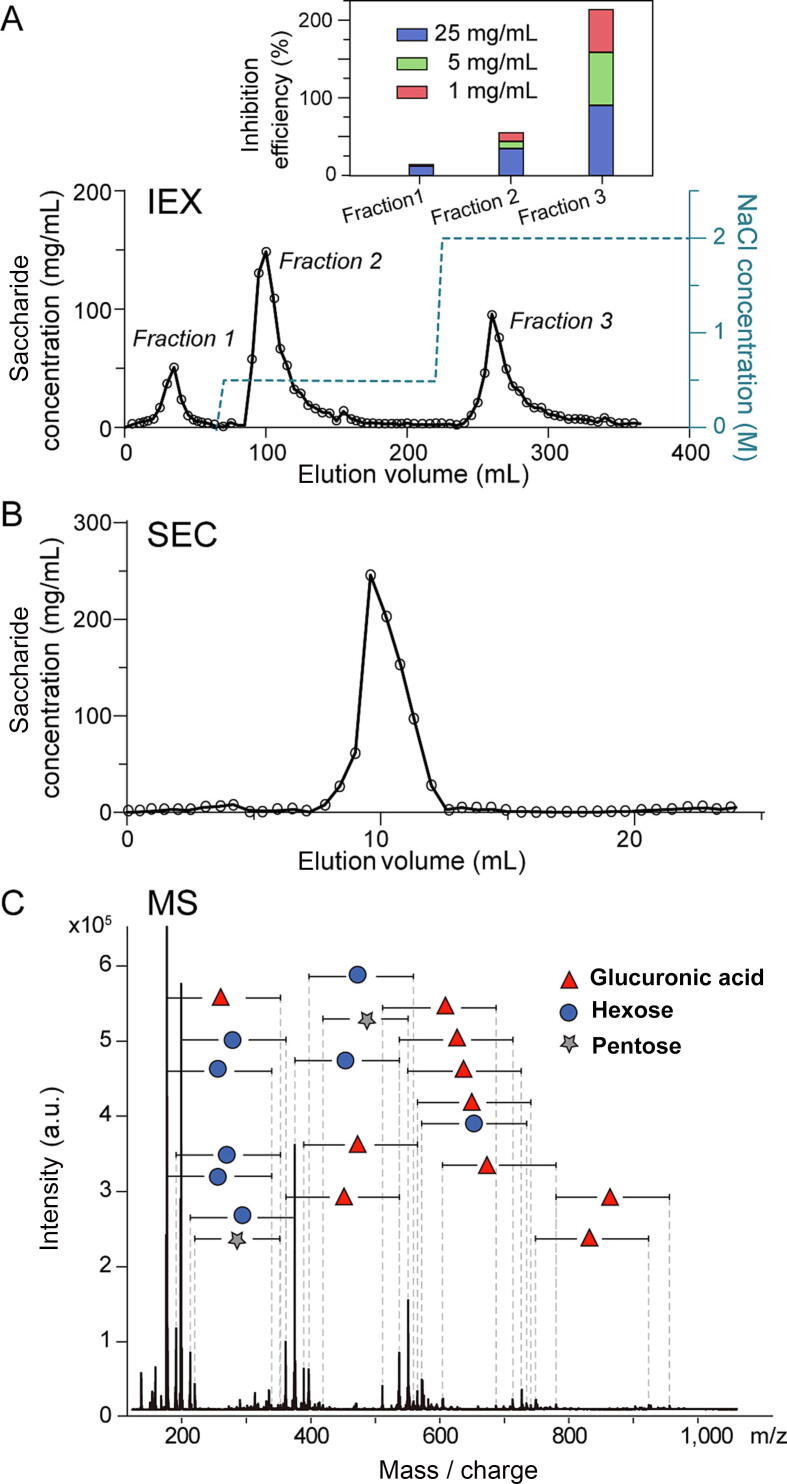
Chromatographic and mass spectrometric characterization of antiviral components from *I. obliquus* extract. A) IEX elution profiles of *I. obliquus* extract and the NaCl concentration gradient in the elute, with the inset depicting the antiviral activity of fractions. B) SEC elution profile of the Fraction 3 of *I. obliquus* extract via IEX. The polysaccharide content was quantified using the phenol–sulfuric acid method. C) MALDI-TOF mass spectrum of the SEC-fractionated *I. obliquus* extract post-acid hydrolysis, revealing the monosaccharide composition of the active macromolecules. Abbreviations: IEX, Ion exchange chromatography; SEC, size exclusion chromatography; MALDI-TOF, matrix-assisted laser desorption/ionization-time of flight; MS, mass spectrometry; a.u., arbitrary unit; *I. obliquus, Inonotus obliquus*.

We performed ion-exchange chromatography (IEX) on extracts obtained from *I. obliquus* to obtain crude fractionation of the extract’s components. We loaded the column with varying concentrations of extracts and subsequently eluted using different concentrations of NaCl (Fig. 3A). Fractions eluted using high-concentration NaCl (Fraction 3, 2 mol/L NaCl elute) exhibited a higher antiviral inhibition efficiency (Fig. 3A inset), indicating that the active components were negatively charged. Given the previous findings that the active components were water-soluble, had a large molecular weight, and were temperature-insensitive, they are likely polysaccharides, which are essential natural products produced by many fungi.

The result of SEC revealed that fractions containing active compounds exhibited a relatively narrow distribution of molecular weight (Fig. 3B), in agreement with the centrifugal fractionation results. To verify the chemical nature of these active compounds, we performed a MALDI-TOF MS analysis. The high-molecular-weight compounds, which were not detectable at the intact level in MS, gave rise to multiple clusters of signals of singly charged ions after acid treatment (Fig. 3C), indicating that they underwent effective acid hydrolysis and produced small fragments. The adjacent signal peaks in each signal series displayed repeating spacings of 132.1 Da, 162.1 Da, and 176.1 Da, corresponding to the masses of a pentose, a hexose, and a glucuronic acid residue, respectively. These mass patterns reveal that the active macromolecules consist of segments built up by these monosaccharide residues in a repeating manner, thereby confirming that the active compounds are indeed polysaccharides. Given that the cleavage of glycosidic bonds is nonspecific under the chemical hydrolysis condition used in this study, the relative abundances of the monosaccharide residues detected by MS reflect the residue composition of the active polysaccharide components with antiviral activities.

## 4 Discussion

Utilizing the methods described above, we conducted a comprehensive screening and evaluation of natural extracts derived from medicinal fungi for their inhibitory activity against SARS-CoV-2 infection, and identified the active macromolecules. The hydrophilicity, thermostability, and molecular weight of the active molecules from *I. obliquus* and *P. adiposa* extracts were evaluated through heat treatment, ethanol precipitation, and molecular weight filtration. The chromatographic and mass spectrometric characterization indicates that the active antiviral molecules are polysaccharides. Our screening approach integrated multiple dimensions of information about the antiviral mechanisms of different natural compounds. While characterizing the inhibition mechanism of these molecules is a complex task and necessitates further investigation, our findings underscore that polysaccharides from medicinal fungi are the principal contributors to the antiviral activity of the screened extracts. The high antiviral efficiency and biosafety of these screened extracts suggest their significant potential for development into an anti-COVID-19 drug. Considering that *I. obliquus* and *P. adiposa* have been consumed by humans for a long time, their aqueous extracts could serve as promising sources of natural antiviral products.

Saccharide-protein interactions are prevalent and often play a crucial role in various biological processes [29], [30]. Synthetic saccharides have been fabricated to control biological functionalities and have emerged as therapeutic strategies for glycan-related diseases [31], [32], [33]. The antiviral actions of polysaccharides could be attributed to the mechanisms that include reducing the ability of viruses to infect cells and multiply through direct impact on the virus surface by the polysaccharides’ negative charge, inhibition of viral adsorption by interacting with virions or virus receptors on the host cell surface, inhibition of virus internalization and uncoating by blocking the allosteric process of virus particles, inhibition of virus transcription and replication through interference with viral replication enzymes or other intracellular targets, and improvement of host immune response through activation of NK cells and macrophages or stimulation of the generation of antiviral immune factors [34], [35]. The structural complexity of fungal polysaccharides provides a multitude of potential sites for blocking protein binding. The workflow employed in this study allows for the screening of fungal polysaccharides with higher inhibition efficiency of interaction between the RBD of SARS-CoV-2 and hACE2. RBD-hACE2 binding may be destabilized by the polysaccharides through conformational rearrangements within the positive patch on the RBD surface and long-range electrostatic repulsion that results in unfavorable interactions with the low-pI ACE2 molecules [36]. The subsequent structural characterization provides implications for their potentially additional antiviral actions through other mechanisms.

Our study diverges from previous reports on natural products that inhibit SARS-CoV-2 infection by highlighting the pivotal role of polysaccharides in the screened extracts. We posit that polysaccharides derived from natural fungi represent a promising source of antiviral macromolecules and show potential for development as safe anti-COVID-19 drugs. While the precise design of glycan structures still requires further significant effort, screening and structural characterization of natural polysaccharides could facilitate the identification of promising candidates.

## 5 Conclusion

In this study, we devised a workflow that integrates viral infection inhibition assays at both cellular and molecular levels, along with molecular separation and characterization, to screen and identify natural products exhibiting antiviral activity. Utilizing this workflow, we screened 167 extracts from 36 medicinal fungi under optimized extraction conditions and procedures for their antiviral activity against SARS-CoV-2. The cell infection assay and viral RNA quantification allowed screening of the inhibitory effects of the extracts on SARS-CoV-2 infection. Their antiviral activities were further validated through an assay that tested the blocking of binding between the viral Spike protein and the hACE2 receptor. Fractionation studies of *I. obliquus* extracts, which demonstrated the most potent antiviral activity among the candidate fungi extracts, were conducted through centrifugal filtration and chromatographic elution in IEX and SEC modes. The results indicated the charging and size properties of the active components, thereby providing insight into their chemical nature. The characterization of the fractionated compounds not only verified these active compounds were polysaccharides but also determined their residue compositions, providing valuable structural information that could be instrumental in drug development. Our findings suggest that natural macromolecules such as polysaccharides derived from certain fungi can serve as potent inhibitors of SARS-CoV-2 infection and RBD-hACE2 binding. These insights underscore the antiviral potential of natural products from medicinal fungi and pave the way for further discovery of novel active molecules with therapeutic potentials.

## Ethics statement

The animal experiments in this study were conducted in compliance with approved protocols and guidelines from the Institutional Animal Care and Use Committee of Beijing Viewsolid Biotechnology Co. LTD (Number: VS2126A00150).
