# Supplementary material for: Screening and identifying natural products with SARS-CoV-2 infection inhibitory activity from medicinal fungi
Source: Biosaf Health. 2023 Dec 31;6(1):12–20. doi: 10.1016/j.bsheal.2023.12.006 (PMC11894996; doi:10.1016/j.bsheal.2023.12.006)
Supplement: Supplementary data 1 [file mmc1.docx]

**Support Information for Manuscript**

**“Screening and Identifying Natural Products with** **SARS-CoV-2 Infection Inhibitory Activity from Medicinal Fungi”**

Shuang Zhao^a,b,1^, Amelia Siqi Huang^c,1^, Weibo Zhang^c^, Lili Ren^d, e^, Hexiang Wang^f^, Jianbin Wang^g,h,*^, Xinyang Shao^i,*^, Guanbo Wang^j,k,*^

*a Institute of Agri-Food Processing and Nutrition, Institute of Plant Protection, Beijing Academy of Agriculture and Forestry Sciences, Beijing 100097, China*

*b Beijing Key Laboratory of Fruits and Vegetable Storage and Processing, Key Laboratory of Vegetable Postharvest Processing, Ministry of Agriculture, Beijing 100097, China*

*c Dalton Academy, The Affiliated High School of Peking University, Beijing 100190, China*

*d NHC Key Laboratory of Systems Biology of Pathogens and Christophe Mérieux Laboratory, Institute of Pathogen Biology, Beijing 100730, China*

*e Key Laboratory of Respiratory Disease Pathogenomics, Chinese Academy of Medical Sciences and Peking Union Medical College, Beijing 100730, China*

*f State Key Laboratory for Agrobiotechnology and Department of Microbiology, China Agricultural University, Beijing 100193, China*

*g School of Life Sciences, Tsinghua University, Beijing 100084, China*

*h Beijing Frontier Research Center for Biological Structure, Tsinghua University, Beijing 100084, China*

*i Changping Laboratory, Beijing 102206, China*

*j Biomedical Pioneering Innovation Center (BIOPIC), Peking University, Beijing 100871, China*

*k Institute for Cell Analysis, Shenzhen Bay Laboratory, Shenzhen 518132, China*

^1^ These authors contributed equally to this work.

* Corresponding authors:

Prof. Dr. Jianbin Wang, E-mail: jianbinwang@tsinghua.edu.cn

Dr. Xinyang Shao, E-mail: xyshao@cpl.ac.cn

Prof. Dr. Guanbo Wang, E-mail: guanbo.wang@pku.edu.cn

**Table of Contents:**

*Supplementary Tables.*..………………………………………………………………………………………………………...… S2-S5

***Supplementary Table***

**Table S1**. Overview of the fungal extracts screened in this work.

|  | Name | Extract condition | Concentration µg/mL |
| --- | --- | --- | --- |
|  | *Agaricus balchaschensis* | water | 1,000 |
|  |  |  | 200 |
|  |  | ethanol | 1,000 |
|  |  |  | 200 |
|  | *Agaricus bisporus* | water | 1,000 |
|  |  |  | 200 |
|  |  | ethanol | 1,000 |
|  |  |  | 200 |
|  | *Agrocybe cylindracea* | water | 1,000 |
|  |  |  | 500 |
|  |  |  | 200 |
|  |  | ethanol | 1,000 |
|  |  |  | 500 |
|  |  |  | 200 |
|  | *Artemisia integrifolia* | water | 1,000 |
|  |  |  | 500 |
|  |  |  | 200 |
|  |  | ethanol | 1,000 |
|  |  |  | 500 |
|  |  |  | 200 |
|  |  | Ethyl ether | 1,000 |
|  |  |  | 500 |
|  |  |  | 200 |
|  | *Auricularia polytricha* | water | 1,000 |
|  |  |  | 200 |
|  |  | ethanol | 1,000 |
|  |  |  | 200 |
|  | *Clitocybe maxima* | water | 1,000 |
|  | *Cordyceps militaris* | water | 1,000 |
|  |  |  | 500 |
|  |  |  | 200 |
|  |  | ethanol | 1,000 |
|  |  |  | 500 |
|  |  |  | 200 |
|  | *Cordyceps sinensis* | water | 1,000 |
|  | *Cordyceps cicadae* | water | 1,000 |
|  |  |  | 200 |
|  |  | ethanol | 1,000 |
|  |  |  | 200 |
|  | *Cryptoporus volvatus* | water | 1,000 |
|  |  |  | 200 |
|  |  | ethanol | 1,000 |
|  |  |  | 200 |
|  | *Dictyophora indusiata* | water | 1,000 |
|  |  |  | 200 |
|  |  | ethanol | 1,000 |
|  |  |  | 200 |
|  | *Engleromyces goetzii* | water | 1,000 |
|  |  |  | 500 |
|  |  |  | 200 |
|  |  | ethanol | 1,000 |
|  |  |  | 500 |
|  |  |  | 200 |
|  | *Flammulina velutipes* | water | 1,000 |
|  |  |  | 200 |
|  |  | ethanol | 1,000 |
|  |  |  | 200 |
|  | *Ganoderma lucidum (*Fermentation broth*)*  *Ganoderma lucidum* | water  water | 1,000 |
|  |  |  | 1,000 |
|  |  |  | 500 |
|  |  |  | 200 |
|  |  | ethanol | 1,000 |
|  |  |  | 500 |
|  |  |  | 200 |
|  | *Gloeostereum incarnatum* | water | 1,000 |
|  |  |  | 200 |
|  |  | ethanol | 1,000 |
|  |  |  | 200 |
|  | *Grifola frondosa* | water | 1,000 |
|  |  |  | 200 |
|  |  | ethanol | 1,000 |
|  |  |  | 200 |
|  | *Hericium erinaceus* | water | 1,000 |
|  |  |  | 500 |
|  |  |  | 200 |
|  |  | ethanol | 1,000 |
|  |  |  | 500 |
|  |  |  | 200 |
|  | *Hypsizygus marmoreus* | water | 1,000 |
|  |  |  | 200 |
|  |  | ethanol | 1,000 |
|  |  |  | 200 |
|  | *Inonotus obliquus* | water | 1,000 |
|  |  |  | 500 |
|  |  |  | 200 |
|  |  | ethanol | 1,000 |
|  |  |  | 500 |
|  |  |  | 200 |
|  | *Lentinus edodes* | water | 1,000 |
|  |  |  | 500 |
|  |  |  | 200 |
|  |  | ethanol | 1,000 |
|  |  |  | 500 |
|  |  |  | 200 |
|  | *Morchella esculenta* | water | 1,000 |
|  |  |  | 500 |
|  |  |  | 200 |
|  |  | ethanol | 1,000 |
|  |  |  | 500 |
|  |  |  | 200 |
|  | *Omphalia lapidescens* | water | 1,000 |
|  |  |  | 500 |
|  |  |  | 200 |
|  | *Oudemansiella apalosarca* | water | 1,000 |
|  |  |  | 200 |
|  |  | ethanol | 1,000 |
|  |  |  | 200 |
|  | *Phellinus igniarius* (cultivated on bags)  *Phellinus igniarius* (cultivated on logs) | water  water | 1,000 |
|  |  |  | 1,000 |
|  |  |  | 500 |
|  |  |  | 200 |
|  |  | ethanol | 1,000 |
|  |  |  | 500 |
|  |  |  | 200 |
|  | *Pholiota adiposa* | water | 1,000 |
|  |  |  | 200 |
|  |  | ethanol | 1,000 |
|  |  |  | 200 |
|  | *Pleurotus citrinopileatus* | water | 1,000 |
|  |  |  | 500 |
|  |  |  | 200 |
|  |  | ethanol | 1,000 |
|  |  |  | 500 |
|  |  |  | 200 |
|  | *Pleurotus eryngii* | water | 1,000 |
|  |  |  | 200 |
|  |  | ethanol | 1,000 |
|  |  |  | 200 |
|  | *Pleurotus nebrodensis* | water | 1,000 |
|  |  |  | 200 |
|  |  | ethanol | 1,000 |
|  |  |  | 200 |
|  | *Pleurotus ostreatus* | water | 1,000 |
|  |  |  | 200 |
|  |  | ethanol | 1,000 |
|  |  |  | 200 |
|  | *Ramaria botrytoides* | water | 1,000 |
|  |  |  | 200 |
|  |  | ethanol | 1,000 |
|  |  |  | 200 |
|  | *Shiraia bambusicola* | water | 1,000 |
|  |  |  | 200 |
|  |  | ethanol | 1,000 |
|  |  |  | 200 |
|  | *Stropharia rugosoannulata* | water | 1,000 |
|  |  |  | 500 |
|  |  |  | 200 |
|  |  | ethanol | 1,000 |
|  |  |  | 500 |
|  |  |  | 200 |
|  | *Trametes robiniophila* | water | 200 |
|  |  |  | 50 |
|  |  | ethanol | 200 |
|  |  |  | 50 |
|  | *Tremella fuciformis* | water | 1,000 |
|  |  |  | 200 |
|  |  | ethanol | 1,000 |
|  |  |  | 200 |
|  | *Tricholoma matsutake* | water | 1,000 |
|  |  |  | 200 |
|  |  | ethanol | 1,000 |
|  |  |  | 200 |
|  | *Tuber melanosporum* | water | 1,000 |
|  | *Volvariella volvacea* | water | 1,000 |
|  |  |  | 200 |
|  |  | ethanol | 1,000 |
|  |  |  | 200 |
